# Association between bisphenol exposure and polycystic ovary syndrome risk: an integrated systematic review and meta-analysis

**DOI:** 10.3389/fendo.2026.1859611

**Published:** 2026-06-11

**Authors:** Zongying Gui, Huizhen Lin, Chuhan Wang, Yingsha Yao

**Affiliations:** Department of Gynecology, Ningbo No.2 Hospital, Wenzhou Medical University, Ningbo, China

**Keywords:** bisphenol A, bisphenol S, endocrine-disrupting chemicals, meta-analysis, polycystic ovary syndrome

## Abstract

**Background:**

Polycystic ovary syndrome (PCOS) is a prevalent endocrine disorder associated with environmental endocrine-disrupting chemicals, particularly bisphenols (BPs) such as bisphenol A (BPA) and bisphenol S (BPS). BPs may disrupt reproductive and metabolic pathways through estrogenic, anti-androgenic, and obesogenic effects, contributing to the pathogenesis of PCOS. However, the epidemiological evidence remains inconsistent.

**Methods:**

This PRISMA-guided systematic review and meta-analysis evaluated observational studies (cross-sectional, case-control, longitudinal) from PubMed and Web of Science up to December 2024. Seventeen studies involving 3, 010 women were included after screening. The quality of the studies was assessed using the Newcastle-Ottawa Scale. Standardized mean differences (SMDs) with 95% confidence intervals (CIs) were calculated using random-effects models due to high heterogeneity (I² > 50%). Subgroup analyses were conducted to explore the sources of heterogeneity.

**Result:**

The meta-analysis revealed significant positive associations between PCOS risk and serum BPA (SMD = 1.32, 95% CI: 0.83–1.82), urinary BPA (SMD = 2.69, 95% CI: 1.41–3.97), and serum BPS (SMD = 0.26, 95% CI: 0.07–0.46). Heterogeneity was high for BPA (I² = 95–99%) but absent for BPS (I² = 0%). Sensitivity analyses excluding lower-quality studies confirmed the robustness of the findings. Subgroup analyses utilizing the Rotterdam criteria slightly attenuated the associations for BPA. Funnel plots indicated no significant publication bias.

**Conclusion:**

Exposure to BPs, represented by BPA and BPS, was significantly associated with an increased risk of PCOS, likely mediated by endocrine disruption, metabolic dysregulation, and epigenetic mechanisms. These findings underscore the necessity for regulatory policies aimed at reducing population-level exposure to BPs, the clinical integration of BPs biomonitoring in the management of PCOS, and further research on gene-environment interactions and non-monotonic dose effects. Addressing exposure to BPs may offer novel preventive and therapeutic strategies for PCOS.

**Systematic Review Registration:**

https://www.crd.york.ac.uk/prospero/, CRD42024578174.

## Introduction

Polycystic ovary syndrome (PCOS) is a prevalent hormonal and metabolic disorder affecting women during their reproductive years, with global estimates of prevalence between 11% and 13% (1). Key characteristics of PCOS consist of elevated androgen levels, irregular or absent ovulation, and specific ovarian structures marked by cyst formation. This syndrome is often linked to several complications such as insulin resistance, obesity, and metabolic syndrome. The diversity of PCOS is significant, and a thorough understanding of its underlying causes is still lacking (2).

The consensus is that the emergence and progression of the condition stem from a combination of genetic predispositions, environmental influences, lifestyle choices, and factors that interfere with hormonal regulation (3). In terms of air quality, pollutants like particulate matter (PM2.5) and nitrogen oxides can negatively affect granulosa cell activity by initiating oxidative stress responses in the ovaries (4, 5). Additionally, disruptions to natural sleep-wake cycles, often seen in shift work or due to nighttime exposure to artificial lighting, have been linked to PCOS (6).

Environmental endocrine-disrupting chemicals (EDCs) are acknowledged as a significant factor in the development of PCOS. These compounds can both imitate estrogen and display anti-androgen effects, leading to serious health concerns. Notably, bisphenols (BPs), especially bisphenol A (BPA), are a prominent category of EDCs commonly present in plastics (7).

BPs interfere with the balance of female reproductive endocrine systems through various pathways. Their estrogen-mimicking effects arise from their competitive binding to estrogen receptor α. This interaction initiates signaling pathways that disrupt the negative feedback mechanisms of the hypothalamic-pituitary-ovarian (HPO) axis, resulting in irregular luteinizing hormone (LH) pulse frequencies (8). Moreover, *in vitro* research has confirmed the anti-androgenic effects of BPs, demonstrating that BPA can diminish the binding affinity of dihydrotestosterone to the androgen receptor (9). Additionally, BPs contribute to metabolic disruptions, including the enhancement of adipocyte growth, increased insulin resistance, and elevated androgen production by ovarian theca cells (10).

Numerous cross-sectional studies have shown that women with PCOS tend to have elevated levels of BPA in their serum and urine compared to those without the condition, yet the connection remains contentious. The available evidence on the association between BPs and PCOS varies depending on the population characteristics, the specific BPs analyzed, and the diagnostic criteria for PCOS. This study employs a meta-analytic method to explore the relationship between different samples and the various BPs linked to PCOS. The findings are intended to provide significant insights into the reproductive toxicity risks associated with EDCs.

## Methods

This investigation meticulously adhered to the standards outlined by the Preferred Reporting Items for Systematic Reviews and Meta-Analyses (PRISMA) throughout the implementation of the meta-analyses. Importantly, this meta-analysis has been formally registered in the PROSPERO database under the reference number CRD42024578174. Furthermore, every component featured in this review was systematically arranged following the checklist and flowchart from the 2020 version of the PRISMA guidelines (11).

### Search strategy

A thorough investigation was conducted utilizing electronic resources such as PubMed and Web of Science to identify relevant studies published up to December 31, 2024. The research inquiry was meticulously crafted following the PEO framework, which includes elements like Patient/Population/Problem, Exposure, and Outcome. To improve the screening process, various filters were implemented in the search engines whenever feasible. These filters outlined specific requirements, including the selection of peer-reviewed articles, credible journals, English-language publications, access to full texts, and a focus on research involving human participants.

### Study design

This evaluation aimed to thoroughly examine all observational analytical research exploring the connection between BPs exposure and the development of PCOS. The review included various study formats, such as cross-sectional, case-control, and longitudinal designs. We intentionally excluded experimental studies that investigated interventions or other experimental approaches related to PCOS, as our primary emphasis was on observational findings that could shed light on the association between BPs exposure and the incidence or presentation of PCOS.

#### Patient/population/problem

The diverse categories of BPs were not seen as restricting factors; instead, this review emphasizes serum or urinary BPs that are associated with PCOS.

#### Exposure and outcome

Several categories of BPs, such as BPA, bisphenol S (BPS), and other BPs, were recognized as influencing factors for individuals. The diagnostic criteria for PCOS encompass the Rotterdam criteria, standards set by the National Institutes of Health (NIH), along with other additional benchmarks.

### Eligibility criteria and study selection

**Figure 1:**
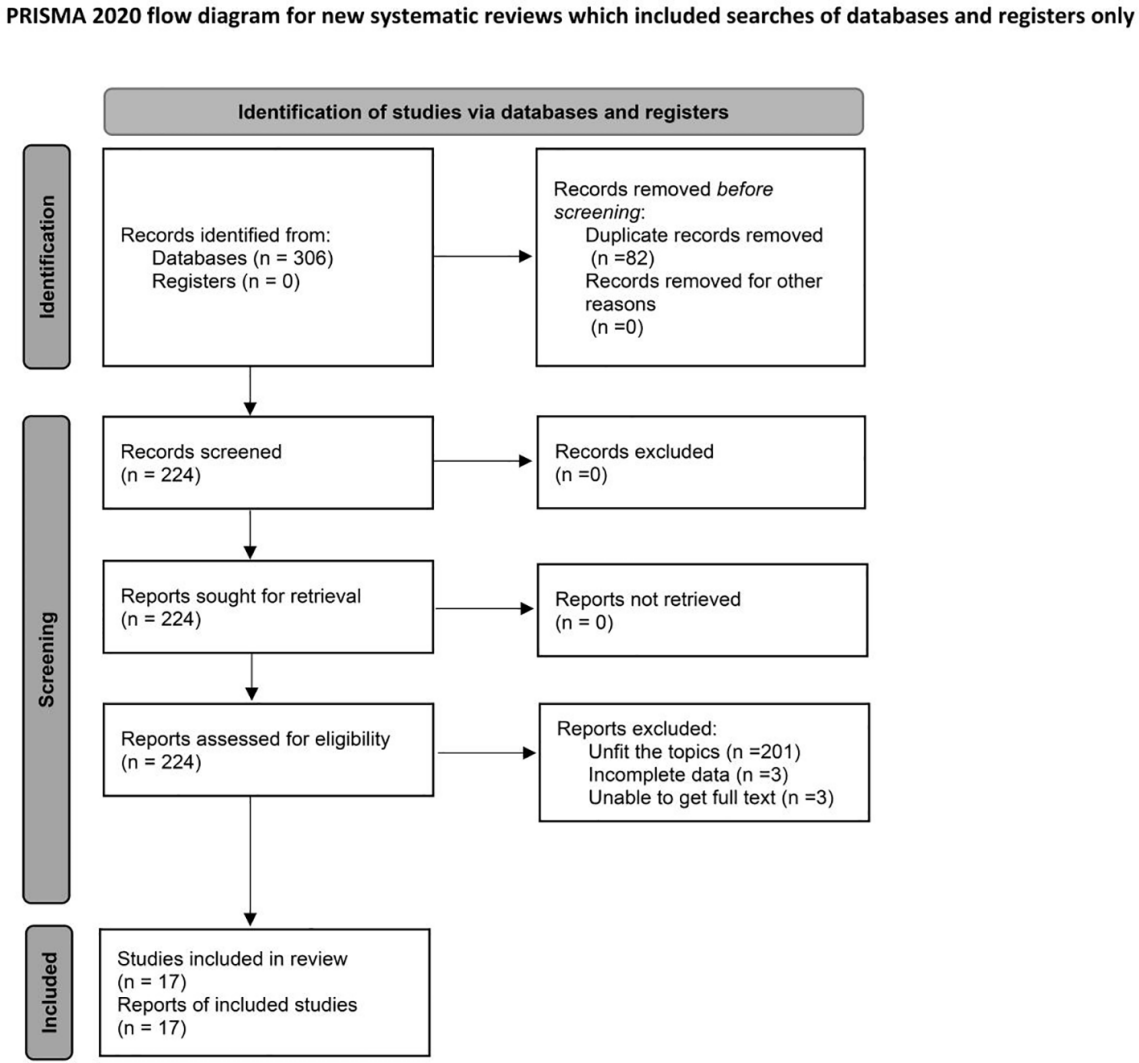
Flow diagram of the study selection process.

Two separate reviewers, referred to as Z.G. and H.L., employed Endnote software (version 20) to thoroughly examine the pertinent records after eliminating any duplicate entries that may have existed. This preliminary measure was essential to maintain the integrity of the dataset. Next, they proceeded to remove additional materials considered outside the scope of the review. The exclusions specifically encompassed letters, books, review articles, conference proceedings, and publications in languages other than English. Moreover, any full texts that were inaccessible and had not been filtered during the initial search refinement were also disregarded. Following this, a detailed review of the full texts of the remaining records was carried out. Throughout this detailed evaluation, articles that did not conform to the designated topics, study designs, or participant criteria defined for the review were removed to ensure a focused selection of relevant studies (see **Figure 1**). During the entire procedure, any disagreements between the reviewers were resolved through constructive dialogue, enabling them to achieve a consensus and affirm the reliability of the review results.

### Assessment of study quality

The main instrument employed to assess the risk of bias in studies that met the eligibility requirements was the Newcastle-Ottawa Scale (NOS) (12). This scale is widely recognized within the academic community as a credible and efficient approach for measuring the methodological integrity of research endeavors. For a thorough analysis, two independent reviewers, Z.G. and H.L., assessed each study for potential bias. In cases where there were differences in the bias assessments, a third reviewer, Y.Y., mediated discussions to address these discrepancies. It is important to mention that research categorized as having a notably high risk of bias was incorporated into the analysis, which aided in developing a more comprehensive understanding of the overall research environment.

### Statistical analysis

The measure of the desired effect size was determined using the Standardized Mean Difference (SMD), which was accompanied by its corresponding 95% confidence interval (CI). To evaluate heterogeneity across the selected studies, we used Cochran’s Q statistic in conjunction with the inconsistency index (I²). When the studies exhibited variations that could affect the results (for example, I² values over 50%), the random-effects model was implemented. Conversely, when there was an indication of minimal variability in the data, we applied the fixed effect model through the inverse variance approach. Additionally, subgroup analyses were performed to pinpoint the sources of heterogeneity, considering factors such as different types of BPs or sample characteristics. To explore the possibility of publication bias, funnel plots were created. All statistical analyses were conducted using Review Manager 5.3 (Cochrane Library), with p-values below 0.05 deemed as statistically significant.

## Results

### Retrieved results

A total of 306 articles were retrieved following a pre-designed literature retrieval strategy; however, this total included 82 duplicates. After examining the titles, abstracts, and full texts, we selected seventeen observational studies that collectively involved 3, 010 women. The process of literature screening and associated results are illustrated in **Figure 1**. The search strategy was (“polycystic ovary syndrome”[MeSH Terms] OR (“polycystic”[All Fields] AND “ovary”[All Fields] AND “syndrome”[All Fields]) OR “polycystic ovary syndrome”[All Fields]) AND (“bisphenol”[All Fields] OR “bisphenolic”[All Fields] OR “bisphenols”[All Fields]).

### Features incorporated into the study

Study Features **Table 1** presents the fundamental characteristics of the studies included in our analysis. These studies were published from 2001 to 2024. Based on the NOS criteria, 13 studies (13–25) were classified as high quality, with 4 studies (26–29) deemed to be of moderate quality.

**Table 1:** Characteristics of included studies.

No.	Study	Type	Country	Years old	PCOS criteria	BPs type	BPs analyses	NOS score
1	Aleksandra Konieczna, 2018	Cross-sectional	Poland	PCOS: 26.9 ± 5.2; Control: 28.2 ± 5.7	AE - PCOS	BPA	HPLC-MS/MS	8
2	Batool Hossein Rashidi, 2017	Case-control	Iran	PCOS: 29.80 ± 7.02; Control: 32.96 ± 5.58	Unclear	BPA	HPLC	8
3	Diana Jędrzejuk, 2019	Case-control	Poland	PCOS: 24.6 ± 5.0; Control: 26.9 ± 6.7	Rotterdam	BPA	HPLC-MS	7
4	Eleni Kandaraki, 2010	Cross-sectional	Greece	PCOS: 28.45 ± 4.80; Control: 32.43 ± 5.35	NIH	BPA	ELISA	7
5	Jalpa Patel, 2024	Case-control	India	PCOS: 29.16 ± 4.15; Control: 24.34 ± 5.11	Rotterdam	BPA	HPLC	7
6	Joanna Jurewicz, 2020	Case-control	Poland	PCOS: 26.6 ± 5.5; Control: 31.2 ± 6.9	AE - PCOS	BPA, BPS, BPF	HPLC-MS	7
7	Leyla Akın, 2015	Case-control	Turkey	PCOS: 15.4 (15.2–15.7); Control: 14.9 (14.5–15.4)	modified Rotterdam	BPA	HPLC	8
8	Mahjoob Vahedi, 2016	Case-control	Iran	control: 28.56 ± 3.29; PCOS: 29.24 ± 3.11	Unclear	BPA	HPLC	7
9	Markéta ŠIMKOVÁ, 2020	Case-control	Czech Republic	Normal-weight PCOS: 28.9 ± 7.4; Obese PCOS: 29.5 ± 5.8 Healthy controls: 29.9 ± 6.4	NIH and Rotterdam	BPA, BPS, BPF, BPAF	LC-MS/MS	7
10	Navya B. Prabhu, 2023	Case-control	India	PCOS: 26.6 ± 5.3; Control: 31.5 ± 6.4	Rotterdam	BPA	ELISA	6
11	Sinem Akgül, 2019	Case-control	Turkey	PCOS: 15.62 ± 1.29; Control: 16.04 ± 1.59	Rotterdam	BPA	HPLC	7
12	Tarantino G, 2012	Cross-sectional	Italy	PCOS: 27.7 ± 6.8; Control: 26.2 ± 3.9	Rotterdam	BPA	ELISA	6
13	Toru Takeuchi, 2001	Case-control	Japan	PCOS: 25.7 ± 1.4; Control: 28.7 ± 0.7	Unclear	BPA	ELISA	5
14	Toru Takeuchi, 2004	Case-control	Japan	Normal-weight PCOS: 26.5 ± 1.5;Obese PCOS: 24.7 ± 1.9 Normal-weight control: 27.5 ± 0.7; Obese control: 28.8 ± 2.0	Unclear	BPA	ELISA	5
15	Wenqiang Zhan, 2022	Case-control	China	20 – 40	Rotterdam	BPA, BPAP, BPAF, BPB, BPS, BPP, BPZ	HPLC-MS/MS	8
16	Yunyao Luo, 2020	Case-control	China	12 – 44	Rotterdam	BPA	GC-MS	8
17	Zora Lazúrová, 2021	Case-control	Slovakia	19 – 43	Rotterdam	BPA	HPLC-MS	7

HPLC, High-Performance Liquid Chromatography; GC, gas chromatography; MS, mass spectrometry; (NIH); (AE - PCOS).

### Results of the meta-analysis

To evaluate the statistical heterogeneity of our findings, we utilized the Chi-squared test, represented by the I² index. An I² value exceeding 50% indicated substantial heterogeneity among the studies included; consequently, we applied the random effects model to synthesize and examine the results.

**Figure 2:**
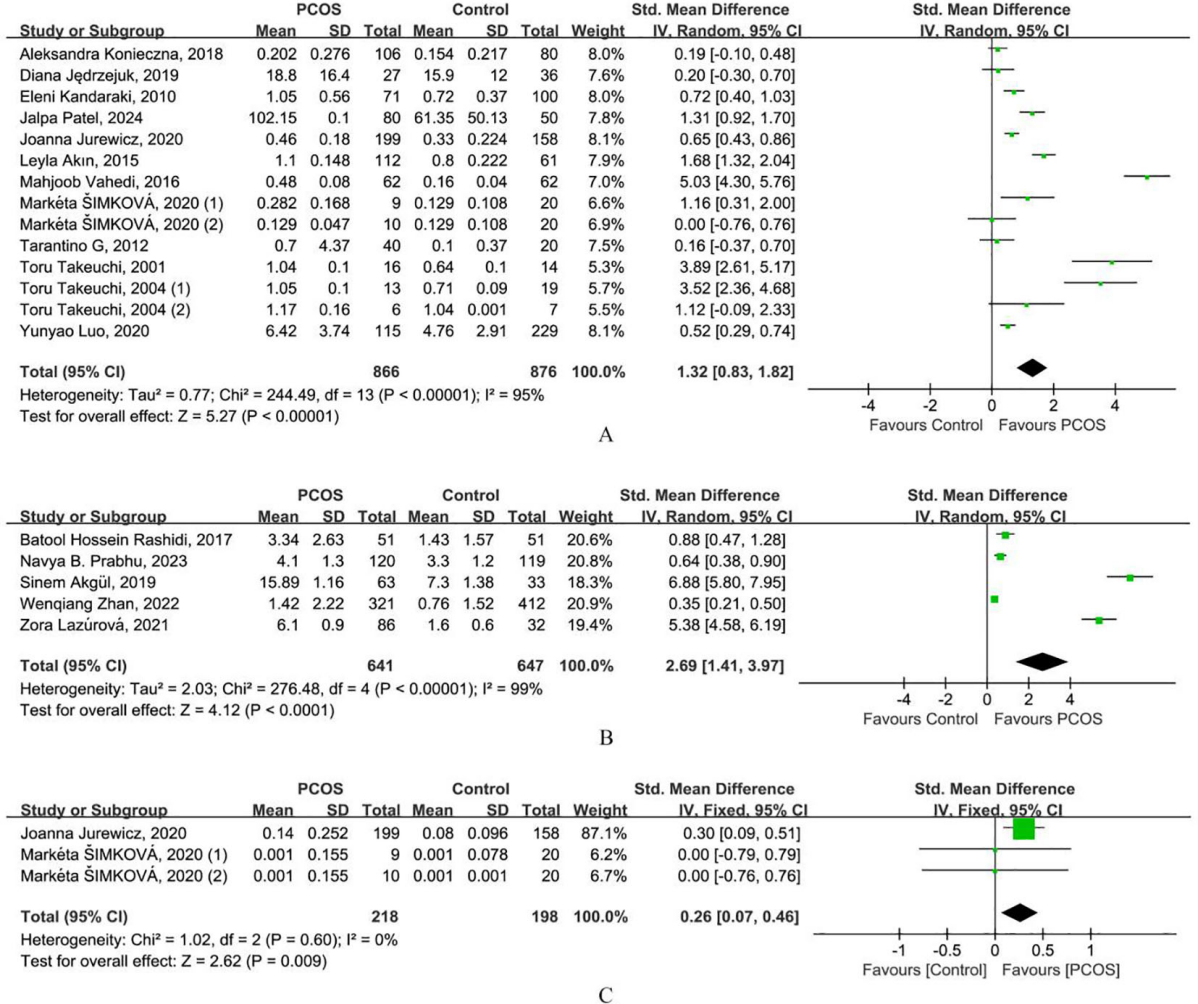
Forest plots for analyzing the relationship between bisphenol exposure and PCOS [**(A)** serum BPA; **(B)** urinary BPA; **(C)** serum BPS].

As depicted in **Figure 2**, our meta-analysis findings indicated a positive correlation between serum BPA, urinary BPA, serum BPS, and the risk of PCOS (pooled SMD: 1.32, 2.69, & 0.26; 95% CI: 0.83 – 1.82, 1.41 – 3.97, & 0.07 – 0.46; I²: 95%, 99% & 0%, respectively).

**Figure 3:**
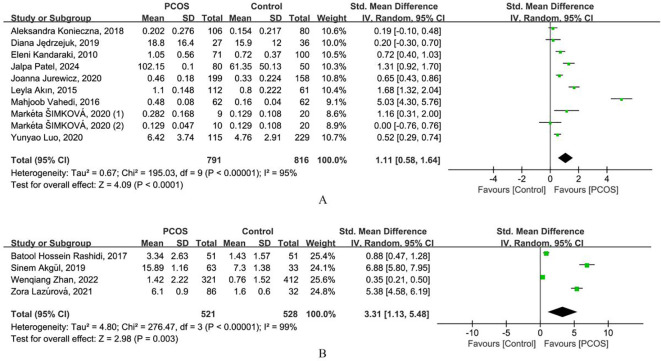
Forest plots for analyzing the relationship between bisphenol exposure and PCOS based on high-quality study data [**(A)** serum BPA; **(B)** urinary BPA].

We expressed concern regarding the presence of four low-quality studies. Nevertheless, the positive correlation observed between BPA and the risk of PCOS remained unchanged after we excluded these two studies, as highlighted in **Figure 3**. The two studies included that investigated BPS and its association with PCOS risk were of high quality.

### Results of the subgroup analysis

**Figure 4:**
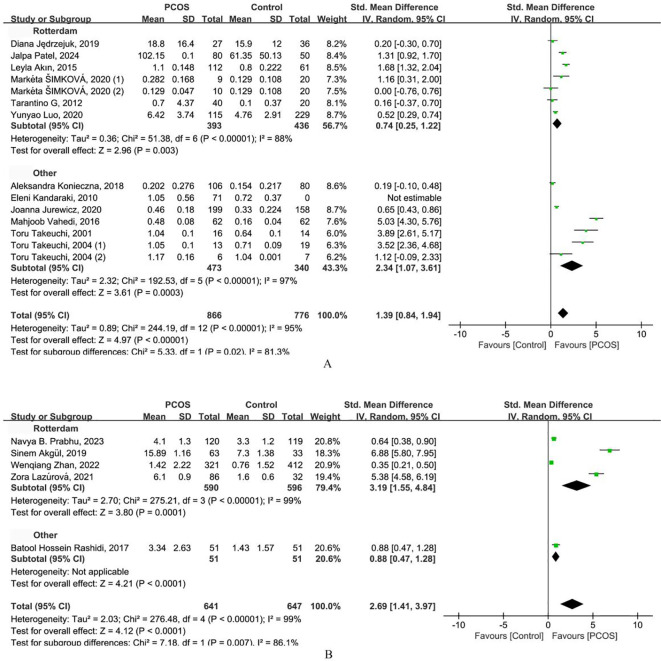
Forest plots for analyzing the relationship between bisphenol exposure and PCOS based on PCOS diagnostic criteria [**(A)** serum BPA; **(B)** urinary BPA].

Taking into account how the diagnostic criteria for PCOS impact the selection of samples, we performed a subgroup analysis of the studies included to determine whether the Rotterdam criteria served as the inclusion standards for PCOS. When focusing solely on the Rotterdam criteria, the pooled SMD values for serum BPA, single BPA, and the risk of PCOS experienced a slight decline. The comprehensive results can be found in **Figure 4**. The outcomes from the subgroup analyses imply that a portion of the observed heterogeneity arises from the studies, yet this does not influence the relationship between BPA and the risk of PCOS.

Moreover, since the two selected studies regarding BPS and the risk of PCOS utilized different diagnostic criteria, this data could not be further examined in subgroups.

### Publication bias

**Figure 5:**
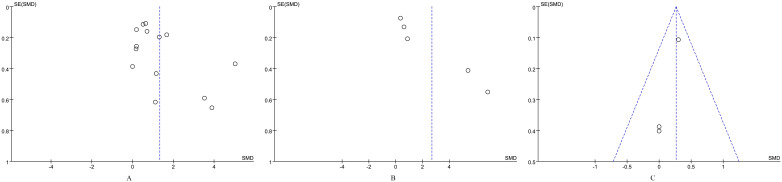
Funnel plots for analyzing the relationship between bisphenol exposure and PCOS [**(A)** serum BPA; **(B)** urinary BPA; **(C)** serum BPS].

We evaluated the presence of publication bias throughout the entire body of literature, encompassing various subgroups. The analyses of the funnel plots revealed no significant evidence of publication bias (refer to **Figure 5**; **Supplementary Figures 1**, **2**).

## Discussion

This comprehensive review and meta-analysis synthesizes data from 17 observational studies involving 3, 010 individuals, revealing a significant association between exposure to BPs and the likelihood of developing PCOS. The BPs under investigation include BPA and BPS. Findings from the meta-analysis demonstrate a positive relationship between serum levels of BPA, specific BPA measurements, serum BPS concentrations, and the risk of PCOS. These findings not only highlight the role of BPs as endocrine disruptors in PCOS development but also emphasize their interactions with genetic, metabolic, and epigenetic factors. Subsequent sections will provide context for these results through an analysis of biological plausibility, explore clinical implications, and propose avenues for future research.

### Biological plausibility of BPs-PCOS association

BPs, including BPA, have the potential to interfere with various endocrine systems and metabolic processes essential for PCOS development, thereby providing support for the observed association.

#### Hyperandrogenism via steroidogenic dysregulation

BPA directly enhances the production of androgens in ovarian theca cells by increasing the expression of CYP17A1 (17α-hydroxylase) and 3β-HSD, which is supported by a 68% rise in testosterone levels seen in granulosa cell cultures that were exposed to BPA (30–32) Animal studies have uncovered a novel mechanism of BPA’s reproductive toxicity by disrupting star-mediated cholesterol transport within mitochondria (33). In terms of mechanisms, the estrogenic effects of BPA activate pathways mediated by ERβ in theca cells, circumventing the typical feedback regulation of the HPO axis (32, 34). In cell culture scenarios, BPS interrupts steroidogenesis at levels that do not cause cytotoxicity, resulting in distinct patterns of hormone levels that are significantly altered (35, 36).

#### Insulin resistance and metabolic disruption

The liver is a primary site for insulin action and also serves as a target organ for bisphenol A. Numerous animal studies have shown that exposure to BPA can influence liver metabolism (37–39). Mice that were treated with BPA exhibited impaired insulin sensitivity in their livers, along with inhibited insulin signaling (40). *In vitro* research has revealed that BPA may induce insulin resistance through the activation of JNK/p38 pathways (41). A strong association has been observed between serum levels of BPA and various biochemical abnormalities—including fasting blood glucose, triglycerides, and HOMA-IR—in patients diagnosed with PCOS (42). Interestingly, BPA’s tendency to promote obesity may be mediated through the activation of PPARγ (43, 44). Nevertheless, the precise molecular mechanisms responsible for BPA-induced insulin resistance and its obesogenic effects are still not fully understood. Prior cellular studies have indicated that exposure to BPS could heighten the risk of insulin resistance via lipid remodeling that involves cardiac lipoprotein, phosphatidylglycerol, and pathways associated with fatty acid metabolism (45).

#### Epigenetic reprogramming

Prenatal exposure to BPA leads to hypermethylation of the LHCGR promoter, which increases the sensitivity of ovarian cells to LH—this is a key characteristic of follicular arrest seen in PCOS (46). Both *in vivo* and *in vitro* transcriptomic studies indicate that BPA may influence TGF-β signaling pathways, independent of the toxicological context applied (47). Research involving transgenerational Medaka Fish models has shown that the offspring of dams exposed to BPA exhibit PCOS-like traits, even in the absence of direct exposure, suggesting the role of epigenetic inheritance through the germline (48). Investigations concerning BPS remain infrequent.

#### Gene-environment interactions

Patients with PCOS often possess variants of the CYP19A1 gene. Exposure to BPA may further impact the ovarian follicles in these individuals, leading to a diminished ovarian reserve. This effect may be linked to the interactions between BPs and the specific genetic variants present in PCOS patients; however, the precise mechanisms involved require additional research (32, 49).

### Public health implications

A general agreement has emerged that BPA is detectable in most individuals within developed nations (50). However, in contrast to BPA, there is no consensus regarding the presence of BPS in the population. Important measures include: 1) Regulatory Policies: The elimination of BPA from thermal paper receipts and food packaging. 2) Clinical Screening: The integration of BPA biomonitoring (such as urinary BPA-glucuronide) into diagnostic evaluations for PCOS in high-risk groups (BMI >25 kg/m² or familial predisposition). 3) Personalized Mitigation: The detoxification of BPA in the liver is reliant on retinoic acidt (51). Individuals with deficiencies in the related enzymatic systems are advised to utilize medical devices that do not contain bisphenol A and to refrain from consuming canned foods (32). Moreover, future studies should focus on human-based cohort designs that assess the effects of BPs exposure dose and duration on PCOS, thereby allowing for the evaluation of potential non-linear dose-response relationships.

### Limitations and methodological considerations

While there is substantial evidence supporting the findings, several limitations necessitate careful interpretation: 1) Residual Confounding: The studies included did not provide information on coexposure to phthalates or organophosphates, which could potentially interact with BPs (52). 2) Exposure Misclassification: Measurements taken from single urine samples only reflect recent exposure, leading to an underestimation of long-term effects (53). 3) Reverse Causality: Hyperandrogenism may impair UGT2B7-mediated clearance of BPA, thus exaggerating the observed correlations (54). 4) Geographic Bias: All studies included in the analysis were conducted in Asia and Europe, which restricts the applicability of the findings to populations with different dietary and regulatory frameworks.

## Conclusion

This study identifies BPs, particularly BPA and BPS, as modifiable environmental risk factors linked to PCOS, operating through mechanisms like endocrine disruption, metabolic alterations, and epigenetic modifications. The robustness and consistency of the evidence call for urgent actions to reduce widespread exposure to these substances, particularly during critical developmental stages. Medical professionals should integrate environmental health evaluations into their PCOS management strategies, while researchers ought to concentrate on elucidating gene-environment interactions and developing specific detoxification strategies. Addressing the intricate role of BPs in the development of PCOS could lead to new avenues for prevention and personalized treatment options for this complex endocrine disorder.

## Data Availability

The original contributions presented in the study are included in the article/**Supplementary Material**. Further inquiries can be directed to the corresponding author.

## Supplementary material

Supplementary Figure 1 Funnel plots for analyzing the relationship between bisphenol exposure and PCOS based on high-quality study data [**(A)** serum BPA; **(B)** urinary BPA].

Supplementary Figure 2 Funnel plots for analyzing the relationship between Bisphenol Exposure and PCOS based on PCOS diagnostic criteria [**(A)** serum BPA; **(B)** urinary BPA].
