# Supplementary figures and images for: Association between bisphenol exposure and polycystic ovary syndrome risk: an integrated systematic review and meta-analysis
Source: Front Endocrinol (Lausanne). 2026 Jun 11;17:1859611. doi: 10.3389/fendo.2026.1859611 (PMC13293787; doi:10.3389/fendo.2026.1859611)

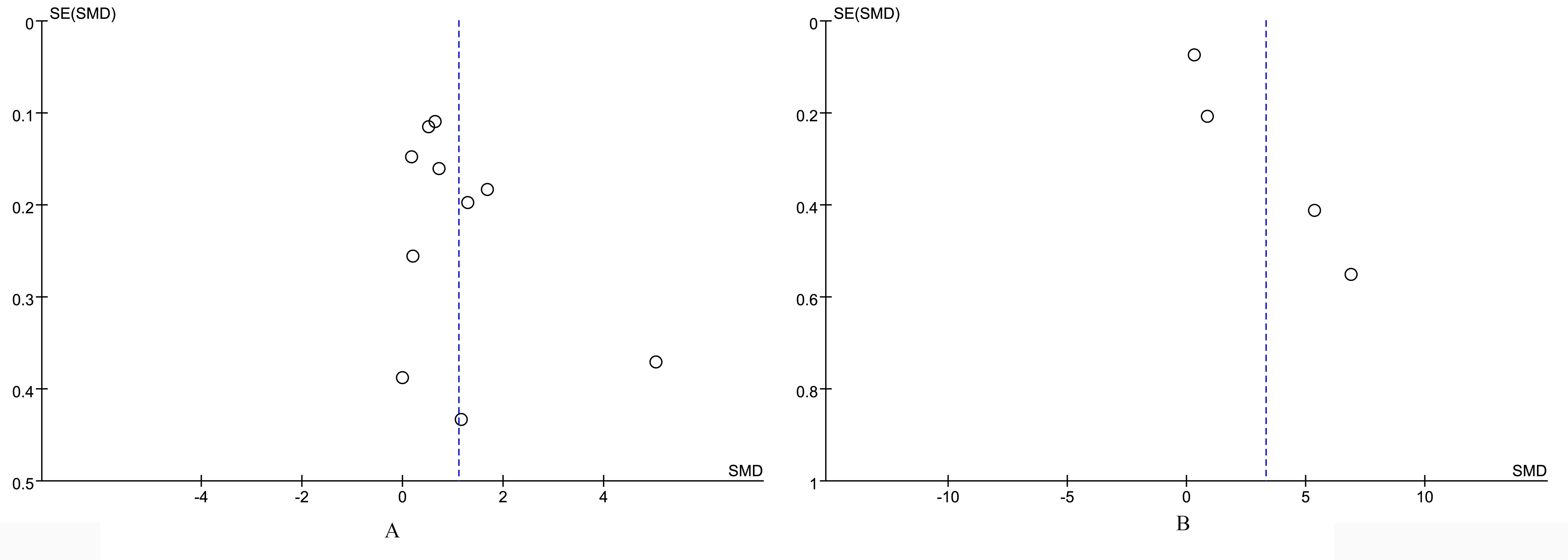

Supplement: Supplementary Figure 1 — Funnel plots for analyzing the relationship between bisphenol exposure and PCOS based on high-quality study data [(A) serum BPA; (B) urinary BPA]. [file SupplementaryFile1.jpg]

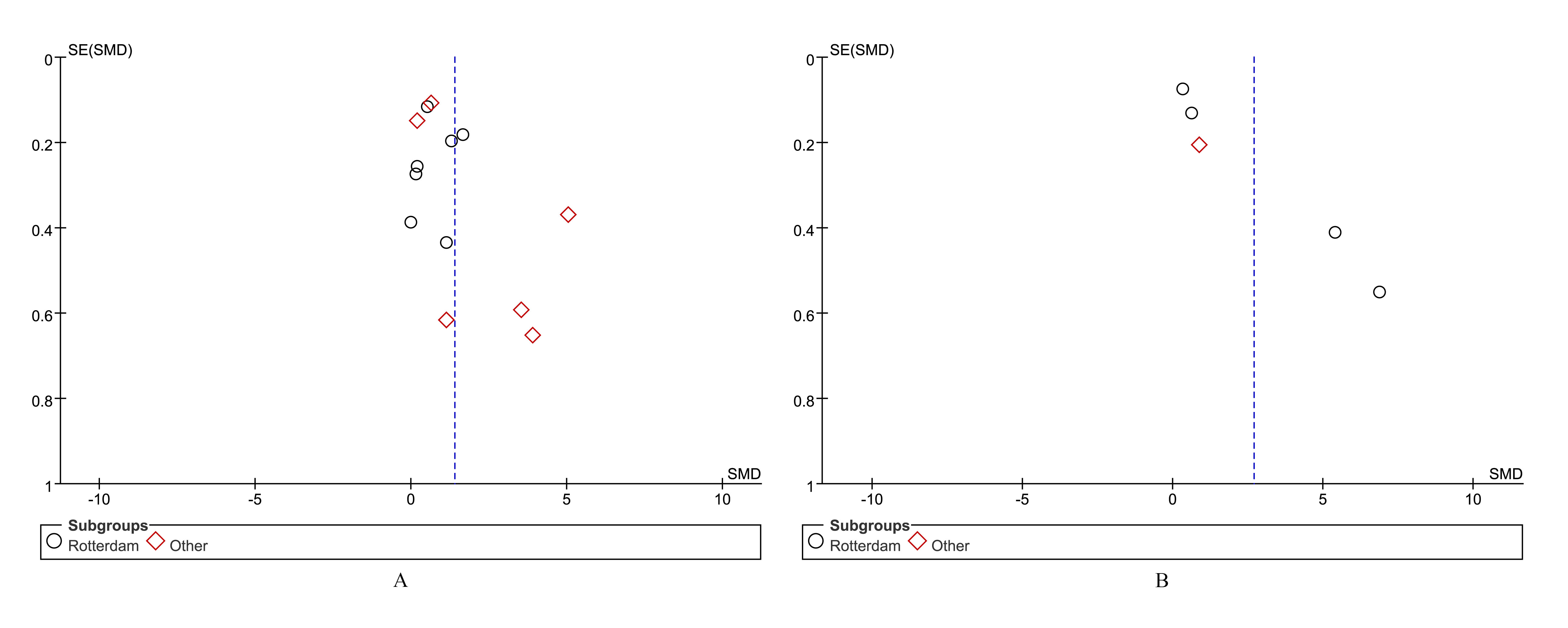

Supplement: Supplementary Figure 2 — Funnel plots for analyzing the relationship between Bisphenol Exposure and PCOS based on PCOS diagnostic criteria [(A) serum BPA; (B) urinary BPA]. [file SupplementaryFile2.jpg]
